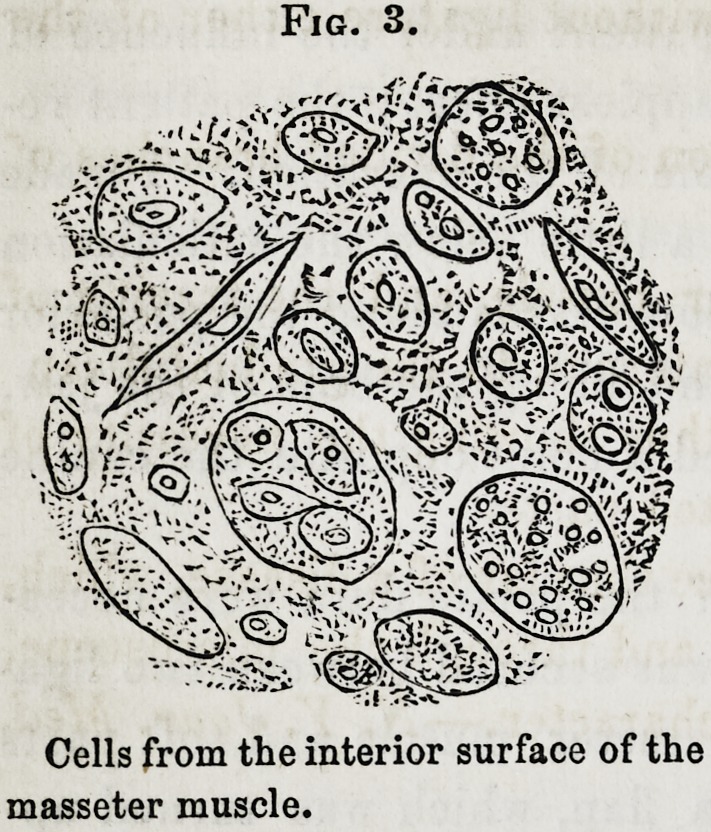# Case of Disarticulation and Removal of Nearly the Lateral Half of the Lower Jaw

**Published:** 1857-07

**Authors:** C. E. Isaacs

**Affiliations:** Demonstrator of Anatomy in the Unversity of New York.


### SELECTED ARTICLES.

### ARTICLE XII.

*Case of Disarticulation and Removal of nearly the Lateral half of the Lower Jaw.* By C. E. Isaacs, M. D., Demonstrator of Anatomy in the University of New York. [With three Illustrations.]

Mr. B., a merchant of this city, residing in Brooklyn, æt. 32 years; of good constitution; in June, 1851, began to complain of some slight pain and uneasiness in the region of the jaw, near the wisdom tooth of the right side, which had not yet made its appearance. In October, 1852, the gum over it was lanced by a dentist, for the first time. During the year 1853, this operation was repeated several times. In December, 1853, it was freely incised by Dr. Bell, of Brooklyn, and some matter was discharged. In October, 1854, Dr. Bell removed a portion of the gum from over the tooth. During the fall of 1854, he had frequent hemorrhage from the part, until December of the same year. Dr. Kissam and Dr. Bell then removed a large portion of the surrounding parts, with the wisdom tooth, which was found lying crosswise, and out of its socket. In January, 1855, a fungous mass, was perceived, to which the nitrate of silver was several times applied, and upon one occasion the actual cautery was used. In March, Mr. B. consulted Dr. N. R. Smith, the distinguished professor of surgery, in Baltimore. He was under his care for about two months.

As the patient resided in Brooklyn, on his return home, Dr.

Fig. 1.

**Appearance of the cancerous mass on the outer surface of the jaw.**

Smith was so kind as to place him under my charge, explaining to me, at the same time, his opinion that the disease was probably of a malignant character. I first saw the patient in the early part of June, 1855, and found, on examination, a fungous mass protruding from the jaw, in the situation formerly occupied by the molar teeth. Observing this carefully for several days, I could not resist the conviction that it was cancerous. I removed a small portion with the scissors, and, placing it under the microscope, found in it abundance of cancerous cells.

I then introduced the patient to Professor Van Buren, without expressing any opinion to him as to its nature; he was decidedly of opinion that it was malignant, and that the removal

FIG. 2.

**Appearance of the cancerous mass on the inner surface of the jaw.—A, cancerous materia protruding from a button-like shell of bone.**

of the jaw was the only chance for the safety of the patient; Dr. Gurdon Buck, who saw the case a few days afterwards, was of the same opinion. Accordingly, I proceeded to operate on the 10th of June, 1855. There were present Drs. Van Buren, Buck, Markoe, Elliot, Gouley, Bell, Catlin, of Youngstown, Niagara Co., N. Y., and Lorette, of Williamsburg. My friend Dr. George T. Elliot placed the patient under the influence of chloroform, which produced the happiest effect; the patient remaining insensible during the whole of the operation. I made a semilunar incision, commencing a little below the articulation of the lower jaw, and extending downwards along the posterior border of the ramus, and under and along the base of the jaw, following its curve until it reached a point opposite the canine tooth.

In this incision, the branches of the portio dura were necessarily divided; the facial artery was secured between two ligatures, and then divided. The masseter muscle and soft parts were dissected up, so as to form a flap, which was turned upwards from the bone, and over the face; the knife was then carried close along the inside of the bone, so as to divide the attachment of the mylo-hyoideus and the mucous membrane of the mouth at a point opposite the first bicuspid tooth, which was then extracted. By the assistance of Drs. Buck and Van Buren, the chain saw was passed around the jaw at this point, and the bone was here divided. The attachment of the internal pterygoid muscle was divided close to the bone; then the dental artery and nerve; by depressing the bone, the insertion of the temporal muscle into the coronoid process was separated; then the attachment of the external pterygoid to the neck of the lower jaw. The capsule was next opened on its external surface; then, by carrying the knife behind the capsule, it, with the remaining parts, was entirely divided, and the disarticulation completed. The hemorrhage was inconsiderable, probably amounting to six or eight ounces of blood.

On looking at the internal surface of the masseter muscle, I noticed it had evidently been contaminated by the disease; I therefore removed the whole of it, and also all the accessible portions of the internal and external pterygoid and temporal muscles, as well as all other parts which had a suspicious appearance, so far as it was safe to do so. After arresting the hemorrhage, and carefully cleaning the surface of the wound, it was brought together by the interrupted suture, adhesive strips, etc., etc.

Fig. 3.

Cells from the interior surface of the masseter muscle.

I was much assisted in the dressing by my friend Dr. Bell, who was so kind as to take charge of the patient for me after the operation, and to his skillful care and attention I attribute much of its success. I examined the inner surface of the masseter muscle, after the operation, and found it filled with cancer cells; I then cut off a thick slice of the inner surface, and, on examining the remaining portion of the muscle from which the slice had been so removed, found but few cells. On making successive slices, the cells were found less and less numerous, until, on approaching the outer surface of the muscle, no cells could be discovered. The wound, throughout a great portion of its extent, united by first intention, but was not entirely healed until October; no unfavorable symptom having occurred during his convalescence.

For many months there was partial paralysis of the branches of the portio dura which had been divided, in consequence of which the muscles were drawn to the opposite side, and it was difficult to close the eyelids. It is now nearly twenty months since the operation. I have this day seen the patient, who is in excellent health, and has gained in weight several pounds. The appearance of the parts in and about the mouth is perfectly healthy.

The restoration of the function of the divided branches of the portio dura has been so perfect that the eyelids of the right side can be as well closed as those of the left; and it would be difficult to trace any difference in the expression on either side of the face. He has raised a large pair of whiskers, which entirely conceal the cicatrix. Much to my surprise, he told me that he could masticate any kind of food almost as well as before the operation.

I may now allude to the most interesting points in the history of this case:

1. The extirpation of the jaw without ligature either of the common or external carotid.

2. The restoration of the function of the divided branches of the portio dura.

3. The removal of the masseter muscle, and the tracing of the cancerous deposit from its internal, to its gradual disap pearance near its outer surface, thus showing the necessity of complete extirpation of all suspected parts.

4. The fact of the non-recurrence thus far of a disease, which, as viewed both by the naked eye and through the microscope, was undoubtedly of a cancerous character.—*N. Y. Jour. Med.*

## Figures and Tables

**Fig. 1. f1:**
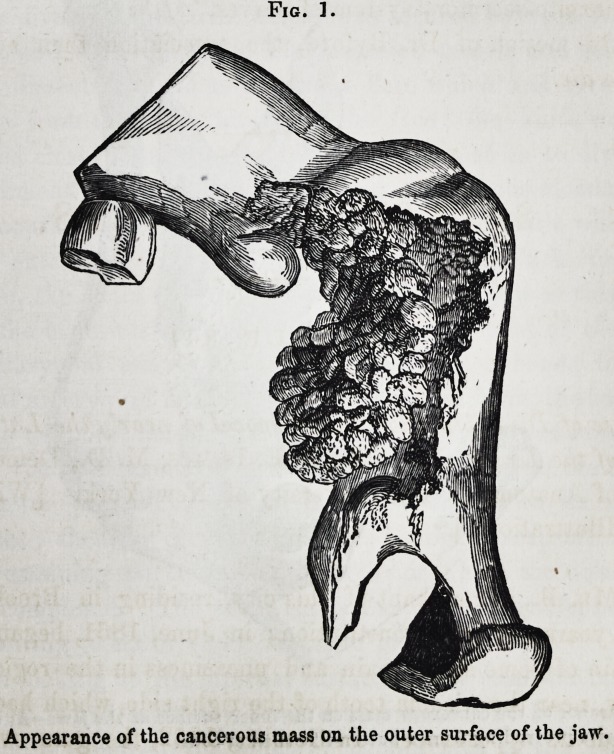


**Fig. 2. f2:**
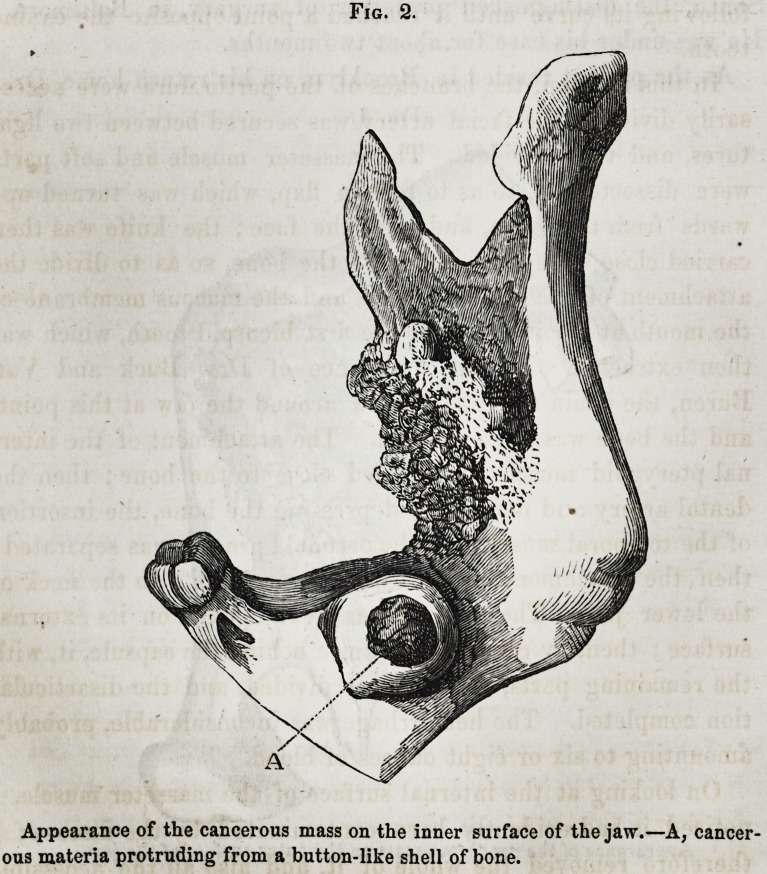


**Fig. 3. f3:**